# Hybrids of the bHLH and bZIP Protein Motifs Display Different DNA-Binding Activities *In Vivo* vs. *In Vitro*

**DOI:** 10.1371/journal.pone.0003514

**Published:** 2008-10-24

**Authors:** Hiu-Kwan Chow, Jing Xu, S. Hesam Shahravan, Antonia T. De Jong, Gang Chen, Jumi A. Shin

**Affiliations:** 1 Department of Chemistry, University of Toronto, Mississauga, Ontario, Canada; 2 Institute of Biomaterials and Biomedical Engineering, University of Toronto, Toronto, Ontario, Canada; University of Queensland, Australia

## Abstract

Minimalist hybrids comprising the DNA-binding domain of bHLH/PAS (basic-helix-loop-helix/Per-Arnt-Sim) protein Arnt fused to the leucine zipper (LZ) dimerization domain from bZIP (basic region-leucine zipper) protein C/EBP were designed to bind the E-box DNA site, CACGTG, targeted by bHLHZ (basic-helix-loop-helix-zipper) proteins Myc and Max, as well as the Arnt homodimer. The bHLHZ-like structure of ArntbHLH-C/EBP comprises the Arnt bHLH domain fused to the C/EBP LZ: i.e. swap of the 330 aa PAS domain for the 29 aa LZ. In the yeast one-hybrid assay (Y1H), transcriptional activation from the E-box was strong by ArntbHLH-C/EBP, and undetectable for the truncated ArntbHLH (PAS removed), as detected *via* readout from the *HIS3* and *lacZ* reporters. In contrast, fluorescence anisotropy titrations showed affinities for the E-box with ArntbHLH-C/EBP and ArntbHLH comparable to other transcription factors (*K*_d_ 148.9 nM and 40.2 nM, respectively), but only under select conditions that maintained folded protein. Although *in vivo* yeast results and *in vitro* spectroscopic studies for ArntbHLH-C/EBP targeting the E-box correlate well, the same does not hold for ArntbHLH. As circular dichroism confirms that ArntbHLH-C/EBP is a much more strongly α-helical structure than ArntbHLH, we conclude that the nonfunctional ArntbHLH in the Y1H must be due to misfolding, leading to the false negative that this protein is incapable of targeting the E-box. Many experiments, including protein design and selections from large libraries, depend on protein domains remaining well-behaved in the nonnative experimental environment, especially small motifs like the bHLH (60–70 aa). Interestingly, a short helical LZ can serve as a folding- and/or solubility-enhancing tag, an important device given the focus of current research on exploration of vast networks of biomolecular interactions.

## Introduction

We utilized our minimalist design strategy to reduce the size and structural complexity of native transcription factors while maximizing retention of DNA-binding function. We focus on three families of transcriptional activators: basic region/leucine zipper (bZIP), basic helix-loop-helix/leucine zipper (bHLHZ), and basic helix-loop-helix/Per-Arnt-Sim (bHLH/PAS). The straightforward α-helical bZIP motif is an ideal scaffold for design of protein:DNA interactions [1]–[6]. Similarly, the bHLHZ utilizes a dimer of α-helices to bind the DNA major groove [7]–[9]. The bHLH/PAS is predicted to adopt similar DNA-binding structure as the bHLHZ motif, based on sequence similarity [10], as no high-resolution structure exists for the bHLH domain in bHLH/PAS proteins.

**Figure 1:**
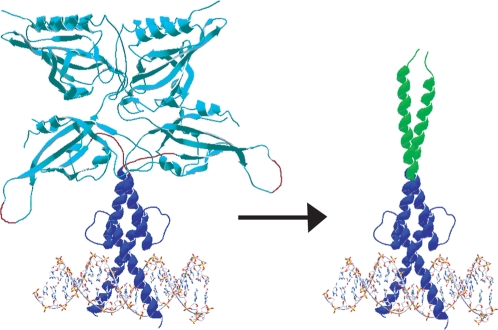
Schematic of minimalist design strategy. By swapping the PAS domain (330 amino acids, teal) of native bHLH/PAS protein Arnt with the much smaller C/EBP leucine zipper (29 amino acids, green helices), a hybrid of the bHLH/PAS and bZIP families was generated and expected to be bHLHZ-like in structure while retaining native Arnt DNA-binding function. Schematic adapted from PDB data. Because no high-resolution structure of an entire bHLH/PAS domain exists, we connected the bHLH domain and PAS domain from different crystal structures and estimated reasonable linkages between the two domains. A single, monomeric PAS A and PAS B repeat was isolated in the Per PAS domain structure (PDB 1WA9) [62]. The dimeric bHLHZ domain in blue (bHLH) and green (leucine zipper) is from the Myc/Max bHLHZ complex with the E-box (PDB 1NKP) [9]. The PAS and bHLH domains are to scale, and we estimated their relative positioning. The orientation of the second identical PAS A/PAS B repeat (two copies of same monomer subunit used) with respect to the Myc/Max bHLH is unknown, and thus we adjusted their orientations to show both structures clearly. Red loops indicate linkages we made by eye.

Proteins containing the bHLH domain, in the presence or absence of additional dimerization elements including leucine zipper (LZ) or PAS domain, can target the Enhancer box (E-box, CACGTG), thereby regulating cellular metabolism, differentiation, and development [11], [12]. In particular, the ubiquitous bHLHZ Myc, Max, and Mad transcriptional activator network serves as a master regulator of the E-box site and is involved in 70% or more of known cancers and tumors [13]. This network is a good starting point for design, for there exists much experimental data including high-resolution structures [7]–[9]. Because of the importance of E-box regulation, we applied our minimalist strategy toward design of simplified proteins that target the E-box based on the bZIP, bHLHZ, and bHLH/PAS scaffolds (Fig. 1): our aim is to generate smaller proteins of simplified structure compared to their native counterparts, while still retaining DNA-binding function. Compared with the native Arnt bHLH/PAS domain at over 400 amino acids (full-length Arnt is almost 1000 aa), our Arnt derivatives comprise 66 or 98 aa, and are therefore accessible by either chemical synthesis or bacterial expression.

Aside from the bHLHZ Myc family, the E-box is also targeted by bHLH/PAS protein Arnt (aryl hydrocarbon nuclear translocator). By heterodimerizing with various partners including AhR (aryl hydrocarbon receptor, also known as the dioxin receptor) and oxygen sensor HIF-1α, Arnt serves as a central regulator in numerous signaling pathways [14]–[16]. Similar to Max, Arnt can also form homodimers that bind to E-box [17], and the Arnt homodimer has been found to activate the transcription of mouse cytochrome P450 2a5, an enzyme involved in the breakdown of toxic substances, including nitrosamines and aflatoxins [18]. The Max homodimer and Myc/Max heterodimer recognize the E-box, and therefore, the Max homodimer may antagonize Myc's cellular functions, including disease-promoting activities [19]. Likewise, the Arnt homodimer, which also targets the E-box, may also interfere with its normal heterodimeric activity [20].

The bHLHZ motif is not as structurally simple as the bZIP, for it utilizes a tetramer of α-helices and an unconserved, flexible loop (HLH) to effect dimerization in addition to its leucine-zipper coiled coil. The bHLH/PAS is even more complicated: the PAS domain comprises 330 aa, which in conjunction with the HLH, is involved in dimerization, structural stability, specification of heterodimerization partner, and ligand binding in response to environmental stimulus [21], [22]. Despite these differences, the basic regions responsible for DNA recognition are highly conserved between the three motifs. Previous studies have shown that within the bZIP or bHLHZ families, basic regions and dimerization domains from different proteins can be exchanged with no change in DNA-binding function [23]–[26].

We therefore extended this notion to exchanging DNA-binding regions and dimerization elements *between* different protein families in order to test our minimalist strategy toward design of hybrid proteins that target the E-box. Our minimalist hybrids were assayed for helical structure by circular dichroism and for E-box binding function both *in vivo* and *in vitro* by yeast genetic assays and quantitative fluorescence anisotropy titrations and compared with previous studies on the AhR/Arnt system [21], [22], [27]. We show that the PAS domain can be replaced by the much smaller leucine zipper to yield a functional DNA-binding hybrid, and that the leucine zipper's main contribution is toward nucleating α-helicity and stability of protein structure.

## Results

Minimalist hybrids of the DNA-binding domain of bHLH/PAS protein Arnt and leucine zipper dimerization domain of bZIP protein C/EBP were designed to target the E-box. By swapping the PAS domain (330 aa) with the much smaller C/EBP LZ (29 aa), a hybrid expected to be bHLHZ-like was generated. Such hybrids test our minimalist design strategy: we hypothesize that we can reduce the size and structural complexity of certain proteins and still retain DNA-binding function. Minimalist hybrids based on the Arnt homodimer may target the E-box and provide a means to modulate E-box regulation. Small proteins that are facile to produce by chemical synthesis or bacterial expression may serve as the basis for design of protein-based therapeutics targeting the Myc:E-box network.

### Three proteins based on Arnt and C/EBP: bHLHZ, bHLH, and bZIP structures

**Figure 2:**
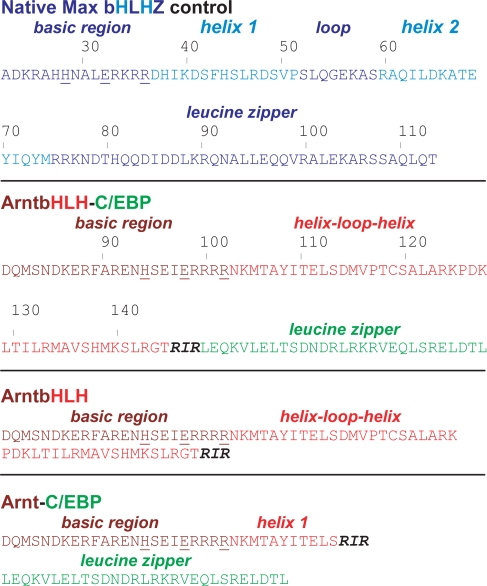
Sequences of hybrid proteins. Max sequences are highlighted in blue, Arnt sequences in red, and C/EBP leucine zipper in green; different shades of blue or red highlight individual components of Max and Arnt. Highly conserved basic-region residues that make sequence-specific contacts to DNA bases in crystal structures are underlined (His28, Glu32, Arg36) [7], [8]. Arnt bHLH components are putative and based on sequence similarity; His94, Glu98, and Arg102 of Arnt aligning with His28, Glu32, and Arg36 of Max are underlined. The nonnative RIR linker is highlighted in bold, black.

To begin our study of how removal or modification of the HLH and PAS domains of Arnt affects its DNA-binding function, three hybrids based on the Arnt homodimer were designed to target E-box. We used the mammalian C/EBP leucine zipper, for it is well characterized and forms a strong homodimer [28]. The first protein, ArntbHLH-C/EBP, comprises the Arnt bHLH domain fused to the C/EBP LZ (Fig. 2); swap of the Arnt PAS for the C/EBP LZ is a dramatic change, for the LZ is one-tenth the size of PAS. Between the bHLH and LZ lies the nonnative RIR linker, which provides a *BamH* I restriction site that facilitates cloning. This construct maintains alignment of the conserved leucines in the C/EBP LZ (Leu/hydrophobic amino acid every seven residues) with Leu142 and Ala135 in Helix 2 of Arnt that may be involved in the hydrophobic interface: hence, this hybrid was intended to mimic the bHLHZ structure of Max, in particular, the alignment of the hydrophobic dimerization interface. Thus, we expected ArntbHLH-C/EBP to be bHLHZ-like, with a seamless α-helix comprising Helix 2 and the C/EBP LZ as shown in the Max bHLHZ homodimer:E-box crystal structure [7], [8].

ArntbHLH-C/EBP is a bHLH/PAS protein converted to bHLHZ. Because the RIR junction between the ArntbHLH and C/EBP LZ is not an optimal sequence for promoting the seamless α-helical structure shown in the crystal structure, as it was introduced for cloning purposes, we hypothesized that although this hybrid would bind to the E-box, its activity could be lower than that of the native Max bHLHZ.

The second protein, ArntbHLH, can dimerize through the HLH domain only, with no LZ or PAS to serve as secondary dimerization domain, akin to native bHLH proteins including MyoD [29]. Utilizing fluorescence anisotropy, Brennan and coworkers demonstrated that their Arnt bHLH domain (56 aa) binds to the E-box with *K*_d_ 56 nM [22]. Chapman-Smith et al. showed that a longer version of the Arnt bHLH domain (142 aa) also shows specific binding to the E-box by electrophoretic mobility shift assay (EMSA) [27]. Thus, the PAS domain is not necessary for the E-box binding function of the Arnt homodimer. Interestingly, these *in vitro* experiments were conducted under low-salt conditions, and both groups observed that the Arnt bHLH domain is sensitive to ionic strength and conditions of experimentation. Given their data, we expected our ArntbHLH to target the E-box site *in vitro* and *in vivo*; we hypothesized that our truncated ArntbHLH might show weaker binding to the E-box than the bHLHZ-like ArntbHLH-C/EBP, which possesses the additional LZ dimerization domain.

The third protein, Arnt-C/EBP, contains the Arnt basic region and a portion of Helix 1 directly fused to the C/EBP LZ: this hybrid lacks the HLH and PAS domains, so the leucine zipper is the only dimerization element. Thus, Arnt-C/EBP is a fusion of bHLH/PAS and bZIP to yield a purely α-helical, bZIP-like protein: this hybrid is the most dramatically changed from native Arnt and the least predictable regarding DNA-binding activity.

### *In vivo* yeast one-hybrid assay

We used the yeast one-hybrid system (Y1H) [30] to examine the ability of the hybrids to activate transcription from the E-box *in vivo*. All hybrid proteins were expressed as fusions with the GAL4 activation domain. We constructed two independent *S. cerevisiae* reporter strains to test the consistency of our results, as assays in yeast can be complicated by false positives [31], [32]. Four tandem copies of the E-box were cloned upstream of either the *HIS3* or *lacZ* reporters, for when flanking sequences between E-box sites were included, background expression was very high requiring >40 mM 3-AT.

We first evaluated activation from the E-box by the *HIS3* reporter assay that allows detection of colony survival under histidine auxotrophy. We then performed two assays based on the *LacZ* reporter: the qualitative X-gal colony-lift filter assay and quantitative *ortho*-nitrophenyl-β-galactoside (ONPG) liquid assay [33]. Though quantitative, the ONPG assay is not sensitive enough to quantify weak interactions accurately [34], so the far more sensitive colony-lift assay is also performed. Although the Y1H does not provide direct detection of binding between our proteins and the E-box, the transcriptional readout of reporter activation generally correlates with protein:DNA binding activity. Hence, the Y1H provides a satisfactory system for *in vivo* testing of protein:DNA interactions.

### The bHLHZ-like hybrid targets the E-box in the Y1H, but the truncated bHLH and bZIP-like hybrid exhibit no activity

**Figure 3:**
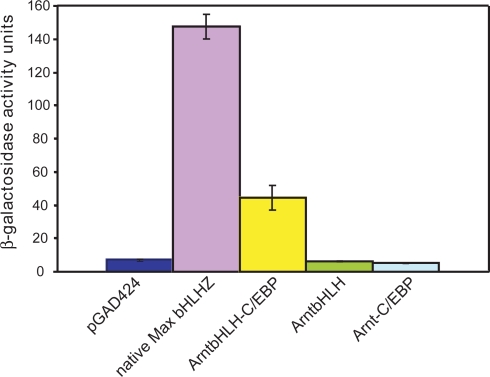
Histogram comparing ONPG assay data. All values are the averages of 9–12 individual measurements from 3–4 separate cell-growth cultures (±SEM).

**Figure 4:**
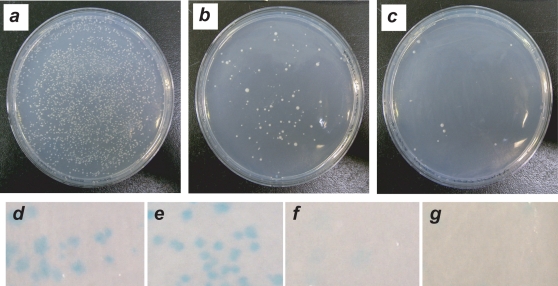
(top) The *HIS3* assay. SD/-His/-Leu plates were incubated at 30°C, six days. Note that bubbles arise from sorbitol in plate medium, and glare is visible in the lower right of each photo. a. Positive control Max bHLHZ on 20 mM 3-AT. b. ArntbHLH-C/EBP on 10 mM 3-AT. c. ArntbHLH-C/EBP on 20 mM 3-AT. (bottom) Colony-lift filter assay. Note that intensity of blue color is affected by variations in colony size. Color intensity in photos is less vivid than actual plates. d. Positive control Max bHLHZ. e. ArntbHLH-C/EBP; positive binding (dark blue). f. ArntbHLH. g. Arnt-C/EBP.

The native Max bHLHZ strongly activated transcription from the E-box in all three assays. We did not generate the native Arnt bHLH/PAS domain (∼400 amino acids), although it binds the E-box [17]. We used the Max bHLHZ (92 amino acids) as a positive control, for it is more similar in structure and size to our designed proteins. This control gives a strong β-galactosidase activity of 147.4±7.3 (Fig. 3). Likewise, the colony-lift assay shows intense blue color; the *HIS3* assay shows strong colony growth at 20 mM 3-AT (Fig. 4), and good colony growth even at 60 mM 3-AT (data not shown). Negative control pGAD424 gives an ONPG reading of 7.0±0.6, with no colony growth by *HIS3* assay and extremely pale color in the colony-lift assay (data not shown). Comparison of the negative and positive controls demonstrates that the GAL4 activation domain alone cannot produce a positive interaction with the E-box site.

The bHLHZ-like first hybrid, ArntbHLH-C/EBP, shows moderate β-galactosidase activity of 44.5±7.4 (Fig. 4). Likewise, *HIS3* colony growth is observed at 20 mM 3-AT, and the colony-lift assay gives bright blue color (Fig. 3). In contrast, the shorter proteins showed no activation from the E-box by any of the above assays: both ArntbHLH and Arnt-C/EBP showed no colony growth on the *HIS3* assay (data not shown), extremely pale color similar to negative control pGAD424 by colony-lift assay (Fig. 3), and β-galactosidase activities of 6.2±0.5 and 5.1±0.3, respectively (Fig. 4).

These *in vivo* data for ArntbHLH are in direct contrast to *in vitro* data showing that the Arnt bHLH domain is capable of targeting the E-box site [22], [27]. Protein:DNA interactions observed in *in vitro* assays are not always reproduced in *in vivo* systems; for example, the deletion mutants of Arnt that showed reduced DNA-binding capability by EMSA failed to exhibit the same DNA-binding in *in vivo* transfection assays [35]. Thus, it is not altogether surprising that the *in vitro* E-box binding of the Arnt bHLH domain observed by Brennan and coworkers [22] and Chapman-Smith et al. [27] cannot be detected in our Y1H system, given that the *in vivo* environment of yeast can vary from chosen *in vitro* conditions.

Transformation of ArntbHLH and Arnt-C/EBP was repeated, and transformants were plated under less stringent conditions (5 mM 3-AT) to ensure that the previous results were not false negatives. As the level of protein expression driven by the truncated *ADH1* promoter in the Y1H system is too low to be detected in the western blot analysis [36], the expression of the GAL4AD-ArntbHLH fusion from pGAD424 in the Y1H was undetectable by western blot. We therefore analyzed ArntbHLH expression in the yeast two-hybrid (Y2H) system, a reporter system similar to the Y1H in this study. In the Y2H system, ArntbHLH is expressed as a fusion to GAL4AD by use of the pGADT7 vector, in which protein expression is under the control of the full-length *ADH1* promoter that leads to a higher level of protein expression. SDS-PAGE and western blot confirmed expression of ArntbHLH in the the Y2H system (Fig. S1, Supporting Information). Given the similarity of the expression cassettes from pGAD424 and pGADT7 (vector information is provided in Fig. S2, Supporting Information), it is unlikely that ArntbHLH is not expressed properly from pGAD424 in the Y1H system.

Because the register of the dimerization element with respect to DNA-binding domain can greatly affect DNA-binding function, we also constructed two derivatives of the bZIP-like Arnt-C/EBP that altered the register of the C/EBP zipper with respect to the Arnt basic region: the last Leu112 and Ser113 in Helix 1 were removed in one derivative, and Ser113 removed in another. Because the α-helix comprises 3.6 amino acids per turn, these three derivatives should provide flexibility in the junction between Arnt and C/EBP to cover all possible orientations of the basic region with regard to the DNA major groove. However, none of the three Arnt-C/EBP proteins could activate transcription from the E-box even after extensive validations.

### *In vitro* fluorescence anisotropy measurements of protein:DNA complexation differ from *in vivo* yeast results

**Figure 5:**
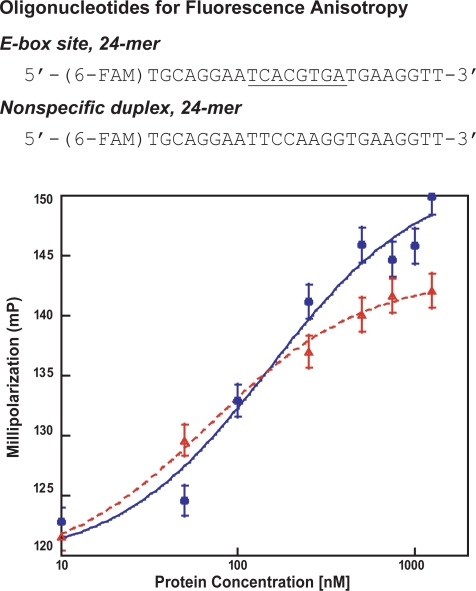
Thermodynamic fluorescence anisotropy titrations. (top) DNA duplexes used in fluorescence anisotropy titrations. “6-FAM” is 6-carboxyfluorescein, and the core E-box is underlined. (bottom) Representative equilibrium binding isotherms for ArntbHLH (▵, dashed red line) and ArntbHLH-C/EBP (•, solid blue line) targeting the E-box. Each isotherm was obtained from an individual titration. Buffer B was used for both titrations. 1.25 µM protein monomer is the highest concentration for which protein solubility is reliably maintained.

The *in vivo* yeast assays measure the ability of our proteins to target the E-box site under the physiological environment of the living yeast cell. However, because yeast reporter assays rely on transcriptional readout for detection of protein:DNA interactions and measurement of binding affinities by ONPG assay is not linear or stringently quantitative [31], we conducted *in vitro* fluorescence anisotropy titrations to measure protein homodimer:E-box dissociation constants. The ArntbHLH-C/EBP and ArntbHLH proteins were expressed and purified from bacteria and assayed with fluorescein-labeled 24-mer DNA duplexes (Fig. 5); no binding by either protein was detected with the nonspecific DNA control, even at 1 µM monomeric protein concentration (data not shown).

We assayed ArntbHLH-C/EBP and ArntbHLH for binding to the E-box in various buffers, for we found that protein:DNA binding activity absolutely depended on conditions of experimentation (see Materials and Methods for details). The high-salt phosphate Buffer A was tried first, as it reasonably mimics a physiological environment, with the addition of 800 mM urea. No reliable fluorescence measurement was obtained for ArntbHLH binding the E-box in Buffer A. We suspect protein misfolding, and possible formation of soluble aggregates, lead to nonfunctional protein, and hence, our use of significant amounts of denaturant that maintains protein solubility, yet decreases the physiological relevance of these experimental conditions. We found protein misfolding and nonfunction to be a more severe problem for ArntbHLH than for its zipper-containing counterpart. For ArntbHLH-C/EBP, weak binding to the E-box could be measured in Buffer A, but these titrations could not be completed, as protein often aggregated at low µM concentrations: from these data, we estimate a *K*_d_ in the high nM range for ArntbHLH-C/EBP binding to the E-box in Buffer A. We therefore tried other conditions, as Buffer A did not provide a reliable environment for obtaining quantitative information.

ArntbHLH-C/EBP displayed less dependence on conditions than did ArntbHLH. ArntbHLH-C/EBP binding to the E-box in the high-salt Tris Buffer B and high-salt phosphate Buffer A discussed above was detectable, and *K*_d_ 148.9±2.9 nM was determined in Buffer B, which contained 200 mM guanidine (Fig. 5). We also obtained good titrations from ArntbHLH binding to the E-box in Buffer B (Fig. 5); interestingly, we measured *K*_d_ 40.2±10.7 nM, which is markedly stronger than that measured for ArntbHLH-C/EBP, and demonstrates that the ArntbHLH effectively targets the E-box. This result is in direct contrast with our Y1H data. ArntbHLH-C/EBP targets the E-box in both the Y1H assay and fluorescence anisotropy measurements, in contrast to ArntbHLH, which shows no E-box binding activity *in vivo* and only under limited conditions *in vitro*.

Our *in vitro* assays of binding of the E-box by ArntbHLH-C/EBP and ArntbHLH were performed in the same high-salt buffer used by Brennan and coworkers in their fluorescence anisotropy titrations [22], with some variations included to improve protein stability (Buffer B; see Materials and Methods). Brennan and coworkers measured a *K*_d_ for the ArntbHLH in complex with E-box in the low µM range, which is much higher than what we measured. Interestingly, when they conducted the titrations in a *low-salt* version of the same buffer, they obtained *K*_d_ 56 nM, which is essentially the same as our measurement of *K*_d_ 40 nM for the ArntbHLH:E-box complex in *high-salt* Buffer B (we remade the buffers to confirm these data). Given the similar experimental conditions and same method of measurement, we conclude that the variant sequences at the N-termini of the different versions and/or the C-terminal 6His tag on our version of ArntbHLH must be the underlying cause of the difference in measured binding affinities (see Materials and Methods for difference in sequences).

We also performed fluorescence anisotropy titrations in Buffer C, which is the identical high-salt buffer used by Brennan and coworkers—i.e. Buffer B without additives that enhance protein folding. We measured identical binding affinities for ArntbHLH and ArntbHLH-C/EBP binding to the E-box: both are approximately 350 nM (see Fig. S3, Supporting Information, for binding isotherms; all isotherms indicate dimeric, cooperative binding with Hill Coefficients of approximately 2). These *K*_d_ values are weaker than those measured in Buffer B, and we suspect that reduced protein stability in Buffer C is responsible for the weaker binding affinities measured.

### Circular dichroism demonstrates that the leucine zipper significantly enhances α-helicity

We hypothesized that the lack of E-box-binding activity of ArntbHLH *in vivo* in yeast must be due to a lack of intrinsically stable structure resulting in protein misfolding and nonfunction, as addition of the C/EBP LZ gives the functional E-box binder ArntbHLH-C/EBP. We note that although both proteins were prone to insolubility in FA, as above, ArntbHLH was far more intractable, and this insolubility may stem from lack of helical, stably folded structure. Thus, we used circular dichroism (CD) to allow comparison of the intrinsic helical structure present in each protein.

**Figure 6:**
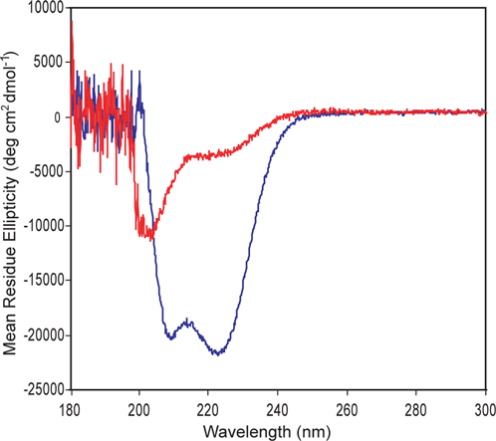
Circular dichroism. Spectra of ArntbHLH (red line) and ArntbHLH-C/EBP (blue line). 2 µM protein monomer was placed in 200 µL Buffer D (15.08 mM Na_2_HPO_4_, 4.92 mM KH_2_PO_4_, pH, 7.4, 50 mM NaCl). Samples were incubated overnight at 4°C, followed by at least 20 min incubation at room temperature. Each spectrum was averaged twice, and curves were not subjected to smoothing. The buffer control was subtracted from each protein spectrum. Mean residue ellipticities are presented, which accounts for differences in lengths of the two proteins.

ArntbHLH-C/EBP is much more helical (56%), and therefore more properly folded and stable, than ArntbHLH, which shows no clear helical structure (Fig. 6). We measured CD under several different buffer conditions, including the presence or absence of urea or nonspecific calf thymus DNA (at the same concentrations used in FA titrations), and consistently found ArntbHLH-C/EBP to possess more intrinsic helicity than ArntbHLH, which showed very little helical, folded structure (see Fig. S4 for CD under other conditions). Addition of urea decreased structure somewhat for both proteins, but did not change the observation that ArntbHLH-C/EBP is strongly helical and ArntbHLH lacks intrinsic structure. We considered whether nonspecific DNA might induce helical structure, in particular, as a means for improving the folded structure of a weakly folded protein like ArntbHLH. Addition of nonspecific DNA increased the helical structure of ArntbHLH, so the presence of the DNA ligand may assist folding and stability in this intrinsically unstructured protein, and hence, the low *K*_d_ value measured for ArntbHLH binding to the E-box. We might surmise that genomic DNA in the yeast cell would also serve the same purpose, but no activation from the E-box by ArntbHLH was detected in the Y1H. Nonspecific DNA decreased helical structure of ArntbHLH-C/EBP, an observation difficult to explain; however, this might explain the weaker binding affinity exhibited by ArntbHLH-C/EBP for the E-box.

Although we cannot assess the structure of ArntbHLH within the yeast cell where we saw no activation from the E-box in the Y1H, the CD conclusively shows that ArntbHLH lacks intrinsic α-helicity and folded structure. This observation supports our interpretation of the discrepancy between the *in vivo* yeast results and *in vitro* FA measurements as being due to nonfunctional ArntbHLH present in the yeast assay. Significantly, the 29 aa C/EBP LZ is sufficient to restore E-box-binding function to ArntbHLH in the yeast environment.

## Discussion

Design of minimal structures is an important starting point in generation of artificial transcription factors. Modulation of specific gene expression can be achieved by small peptides or molecules that mimic native transcription factors, thereby providing applications in diverse fields such as drug discovery and functional genomics [37]–[39]. For example, artificial transcription factors based on the Zn-finger motif have been successfully developed [40]–[43]. By producing functional hybrids from domain swaps between different DNA-binding protein families, we gain insight into how to generate minimalist proteins based on simpler structural motifs that target DNA-binding sites regulated by structurally more complicated motifs. These minimalist hybrids of Arnt and C/EBP are part of our effort to generate proteins with desired DNA-recognition capabilities from a core, α-helical scaffold. Our minimalist hybrids are likely to be easier to express or synthesize in comparison to their native counterparts, as well as to characterize (e.g. high-resolution studies). Thus, they can provide a sound initial platform for protein-based design of new molecules capable of targeting the E-box and modulating the Myc transcription factor network.

### Is protein misfolding the reason for the different results obtained *in vivo* and *in vitro*?

The truncated ArntbHLH domain does target the E-box, as shown by Brennan [22], Chapman-Smith [27], and our group; conditions can be found such that *K*_d_ values in the nM range can be measured for this complex. But this interaction could not be measured in the yeast cellular environment. Only when the leucine zipper was attached to the ArntbHLH was transcriptional activation detected from the E-box site in the yeast one-hybrid assay.

Our observation by *in vitro* fluorescence anisotropy titrations that ArntbHLH targets the E-box more effectively than ArntbHLH-C/EBP is in sharp contrast with our *in vivo* Y1H data. CD shows that the leucine zipper in ArntbHLH-C/EBP can serve to nucleate and stabilize the proper folding of the bHLH domain by initiating α-helix formation, thereby avoiding misfolding and aggregation. Hence, E-box binding activity is observed in the Y1H and in fluorescence studies under more diverse buffer conditions for the more stably folded ArntbHLH-C/EBP than for ArntbHLH.

However, we emphasize that both ArntbHLH and ArntbHLH-C/EBP were difficult to manipulate *in vitro*. For example, in addition to extensive testing of different buffers and salts for the fluorescence titrations, each addition of protein to the sample required *overnight* incubation at 4°C for proper protein folding (see Materials and Methods for details). In comparison, a 2 hr incubation is typically sufficient to achieve stable, soluble protein [44], [45].

Thus, the LZ does not contribute to protein dimerization affinity, as measured by the free energies of the overall protein homodimer:E-box DNA complexes, but rather the LZ encourages a more properly folded, stable bHLH structure capable of DNA-binding function. In related experiments with the ArntbHLH expressed in a different yeast strain (for Y2H analysis), western blot demonstrated that the GAL4 AD fusion of ArntbHLH is present in the soluble fraction after cell lysis (Fig. S1, Supporting Information); our western blot and CD results suggest that misfolding, with perhaps formation of soluble aggregates, is more likely than outright insolubility of ArntbHLH in yeast cells.

While our work was in progress, Chapman-Smith and Whitelaw published their Arnt bHLH-Max LZ hybrid, similar to our ArntbHLH-C/EBP; they also emphasized that their protein constructs (bHLH and bHLH/PAS derivatives) were prone to aggregation, although Arnt-Max was more soluble than their other constructs [21], in parallel to our observations. In particular, their ArntbHLH domain, which contains an N-terminal thioredoxin and 6His tag, was the most intractable, as it had strong tendency for aggregation and was toxic to bacteria during expression and produced low yields [27]. Thus, the authors could not obtain a quantitative *K*_d_, as their Arnt bHLH could not be fully purified due to aggregation at high nM concentrations presumably from improper folding. However, they show by EMSA that their Arnt-Max homodimer binds to the E-box with comparable, but weaker, affinity than does the ArntbHLH/PAS domain [21]. Coincidentally, we, too, made an ArntbHLH-MaxLZ derivative and found that its transcriptional activity closely resembled that of ArntbHLH-C/EBP in the Y1H assays.

We suspect E-box binding by ArntbHLH does not occur in yeast because of protein misfolding and/or aggregation. Both Brennan's and Chapman-Smith's work demonstrated that the Arnt bHLH domain is particularly sensitive to ionic strength; Brennan and coworkers speculate that this sensitivity is due to salt competition at the Arnt dimerization interface, as it is more hydrophilic than other bHLH motifs, which depend on largely hydrophobic dimerization interfaces in the HLH's tetramer of α-helices [22], [27]. Protein misfolding and aggregation appears to be widespread in studies of the bHLH superfamily of transcription factors, as those groups who measured the binding affinity of the Max bHLHZ domain with the E-box site also experienced difficulty with protein aggregation [46]–[48].

### Optimization of the junction between the bHLH and LZ domains may positively affect protein structure and DNA-binding function

The leucine zipper does not contribute positively to the binding affinity between ArntbHLH-C/EBP and the E-box, for its binding affinity is almost 4-fold weaker than that for ArntbHLH. Chapman-Smith and Whitelaw found that that the region between Helix 2 and the PAS domain in Arnt and AhR shows conformational flexibility [21]; by replacing the PAS domain with the C/EBP leucine zipper, our design may have deleteriously altered the flexibility intrinsic to Arnt at the protein-protein interface. Hence, the ArntbHLH-C/EBP dimer may not have the more optimal structure of the native Arnt bHLH/PAS domain for binding the E-box sequence.

We can also compare the E-box-binding affinities of our Arnt derivatives to the native Max bHLHZ domain. By use of EMSA [46], fluorescence anisotropy [47], or calorimetry [48], three groups measured very low nM *K*_d_ values in the 1–3 nM range. Neither the truncated ArntbHLH nor the bHLHZ-like ArntbHLH-C/EBP target the E-box as tightly as does the Max bHLHZ, which might be expected given that the RIR linker at the junction between Helix 2 of ArntbHLH and the C/EBP LZ is not an optimized sequence, as it merely facilitates cloning; Arg does not have strong propensity for forming and stabilizing α-helices [28], and thus, the RIR linker would not be expected to be particularly effective at maintaining the seamless α-helix present in the bHLHZ structure. Our *in vivo* Y1H results also show the same trend as these *in vitro* measurements, for ArntbHLH-C/EBP did not show as strong positive binding signals to the E-box as did the Max bHLHZ control, and the ONPG value for the Max bHLHZ was ∼three-fold higher than that for ArntbHLH-C/EBP.

### The relationship between proper protein structure and detectable DNA-binding function

We tested our minimalist design strategy in the native cellular environment of yeast, and compared these *in vivo* results with *in vitro* quantitative measurements of protein:DNA complexation. In the *in vivo* yeast assay, our results demonstrate that for Arnt, the HLH domain and a second dimerization element LZ are critical for DNA-binding function. However, in *in vitro* fluorescence anisotropy experiments, the ArntbHLH domain is sufficient for strong and specific targeting of the E-box, but only under select experimental conditions. The bZIP-like Arnt-C/EBP, plus the two derivatives with deletions in the junction between the basic region and leucine zipper (discussed above), did not show E-box targeting activity whether *in vivo* in the Y1H or *in vitro* by EMSA (data not shown). A possible reason that the Arnt-C/EBP derivatives, in which the HLH has been removed entirely, showed no ability to target the E-box is that the HLH domain of Arnt may interact with the DNA phosphodiester backbone. In comparison, the Max homodimer:E-box structure shows that residues in the HLH contact the DNA; Lys57 (Loop) and Arg60 (Helix 2) make nonspecific Coulombic contacts to the phosphodiester backbone [7].

By attaching a short, well-folded α-helical appendage, a relatively intractable protein can be converted to a protein amenable to testing under diverse experimental conditions, whether *in vivo* or *in vitro*. We conclude that the C/EBP LZ fused to the ArntbHLH domain does not replace the PAS domain with regard to DNA-binding function; the *K*_d_ value of the truncated ArntbHLH is actually lower than that for its counterpart with the fused zipper. However, the LZ does promote properly folded protein structure, as measured by CD, that is capable of DNA binding and stabilizing folded structure, which is one of the roles that the native PAS domain serves.

Our results also indicate that given the modern focus of exploring vast networks and pathways in genomes, proteomes, and metabolomes, false negative observations may cause true positives to be missed. It was estimated that the percentage of false negatives in a Y2H system used to map protein interactions in *C. elegans* was approximately 45% [49], and in our case, we would have falsely concluded that the ArntbHLH is incapable of targeting the E-box had we been conducting large-scale *in vivo* selections to find protein:DNA complexes. Thus, the presence of false negatives, which can be numerous, is a major issue that needs to be considered.

Our experiments with ArntbHLH and ArntbHLH-C/EBP serve as a cautionary tale, for we started with the yeast reporter assays, and our interpretation of the results dramatically changed once we performed the FA titrations and CD spectroscopy. Possibly, the widespread problem of protein misfolding and aggregation leads to many true positives being skipped. Studies involving vast searches of sequence space may be limited to finding only those molecules that remain soluble and stably folded in a particular assay, and therefore, such examinations will be incomplete. We therefore suggest that in the cases of searches of large libraries, these results be interpreted as specific to a particular assay under specific conditions, and that other results may be obtained from the same library by different assay techniques or even the same technique but under different conditions. Our suggestion does not invalidate previously published “hits” discovered from library searches; on the contrary, we emphasize that other hits may be uncovered as well, and that characterization of hits by different techniques is necessary when interpreting results.

Indeed, many researchers focus their efforts on protein fragments or isolated domains, including library searches or protein design and mutagenesis, as in our case; we often anticipate that these protein fragments will behave well, i. e. assume folded, stable structure and retain significant functional ability. However, this assumption may not always be well-founded: the protein fragment has been removed from its native full-length protein and removed from its native operating environment, both being dramatic changes from its normal context. We often also expect that these shorter, seemingly well-folded structures, such as the α-helical transcription factors examined here, will be folded and stable without assistance from chaperones or heat-shock proteins and in an artifically chosen environment, whether *in vivo* or *in vitro*. As demonstrated here, such long-held assumptions about protein structure and function may lead to a false conclusion, where in fact, the negative observation can be attributed simply to a nonfunctional protein structure under particular experimental conditions.

Perhaps it is easy to view a high-resolution crystal structure, for instance, as *the* protein structure, as we do not actually know how dynamic the protein is, how varied the different conformations are, and how much of the time the protein structure is as the high-resolution picture depicts. Even the seemingly straightforward bZIP structure has proven too dynamic for high-resolution *solution* studies. The GCN4 bZIP basic region is disordered until binding to DNA: both NMR and CD demonstrate that while the leucine zipper is intrinsically stable and helical, the basic region remains only loosely helical until binding to DNA [50]–[54]. In NMR studies on the GCN4 bZIP:AP-1 complex, Palmer and coworkers found that although the GCN4 basic region is substantially helical, it is highly dynamic in the DNA major groove [55]. The only high-resolution structures of the bZIP:DNA complex have, therefore, been obtained by crystallography; we note that the same holds true for bHLH and bHLHZ proteins, as well, likely due to the basic region these motifs share in common. Thus, we conclude that some transcription factor families are highly dynamic, even when bound to the DNA ligand, and their structures cannot be captured by high-resolution solution techniques.

In a recent historical perspective, Alan Fersht emphasized that as much as 40% of proteins in the human proteome are estimated to be intrinsically disordered and may become more or fully folded upon binding their specific cellular ligand [56]: hence, the highly dynamic nature of protein structure. The possibility of Nature using unstable protein structures as a means for performing a wide variety of functions in the cell is not only intriguing, but also highlights the unpredictability inherent in protein research, whether *de novo* protein design or searching libraries of proteins or protein fragments. Given these challenges, Fersht notes that the most effective protein design strategies incorporate what Nature has already devised, but even so, we demonstrate that our design, which is based on native structures, behaved very differently when assayed by various techniques.

As demonstrated here, the discrepancy between *in vivo* and *in vitro* measurements could be clearly ascribed to misfolding of the protein under question; other researchers also reported similar problems with folding and solubility in their Max and Arnt derivatives. Quite often, solubility-enhancing tags are fused to proteins, whether they are being screened *in vivo* or overexpressed for large-scale *in vitro* studies, and these tags can be large. The only difference between ArntbHLH and ArntbHLH-C/EBP is the 29 aa C/EBP LZ, whether expressed in the Y1H or produced by bacterial expression for quantitative examination. Thus, even a small α-helix can enhance protein folding and stability; we used a leucine zipper in these studies, and likely a more hydrophilic, yet well folded, α-helix would serve as a better folding- and solubility-enhancing tag.

## Materials and Methods

### Construction of the *HIS3* Reporter Strain YM4271[pHisi-1/E-box]

100 ng of each of the two complementary 30 bp oligonucleotides, 5′-AATTC CACGTG CACGTG CACGTG CACGTG T-3′ and 5′-CTAGA CACGTG CACGTG CACGTG CACGTG G-3′ with 26 bp overlap underlined, were annealed by heating at 70°C for 5 min (in 50 mM NaCl, 10 µL reaction volume) and slowly cooled to room temperature. The annealed duplex contained four tandem copies of the E-box target sequence (5′-CACGTG) and was cloned into pHISi-1 integrating reporter vector at the *EcoR* I and *Xba* I restriction sites upstream of the *HIS3* reporter gene. After insertion of the E-box sequences, the recombinant pHISi-1 vector was sequenced and linearized at the *Xho*1 site and integrated into the *his3-200* locus (*MATa, ura3-52, his3-200, ade2-101, lys2-801, leu2-3, 112, trp1-901, tyr1-501, gal4-D512, gal80-D538, ade5::hisG*) of *Saccharomyces cerevisiae* YM4271 (Matchmaker One-Hybrid System, Clontech, Palo Alto, CA) to produce the reporter strain YM4271[pHisi-1/E-box]. This reporter strain was selected and maintained using minimal medium plates lacking histidine.

In order to assess background due to leaky His3 expression, 3-aminotriazole (3-AT) was used as a competitive inhibitor of the His3 protein. The reporter strain was titrated on SD/-His plates with varying amounts of 3-AT (0–60 mM) to determine the optimal concentration of 3-AT for background suppression (Matchmaker One-Hybrid System, Clontech). 10 mM 3-AT was sufficient for background suppression in YM4271[pHISi-1/E-box].

### Gene construction for ArntbHLH, ArntbHLH-C/EBP, and Arnt-C/EBP

DNA oligonucleotides were purchased from Operon Biotechnologies (Huntsville, AL). The genes encoding the ArntbHLH (or Arnt basic region with part of Helix 1, as in Arnt-C/EBP) and C/EBP leucine zipper were constructed separately; we used the sequences from human Arnt isoform variant 3 (NCBI NP_848514) and rat liver C/EBP. The genes for expression of C/EBP LZ and Arnt basic region (with portion of Arnt Helix 1) were constructed from two unique oligonucleotides with 21 bp overlap by mutually primed synthesis [45] and amplified with terminal primers by use of the Advantage 2 PCR Kit, following the manufacturer's instructions (Clontech). Gene assembly and amplification were performed in two separate PCR reactions (Thermo Hybaid Sprint). The gene of ArntbHLH was synthesized by the method described by Wu and coworkers [57]; a series of six sequentially overlapping oligonucleotides was assembled, extended, and amplified in a single PCR reaction. Amplified gene inserts were purified by Minelute PCR Purification Kit (Qiagen, Mississauga, ON). The gene for the C/EBP LZ was inserted into the *BamH* I and *Pst* I sites of vector pGAD424 (Matchmaker One-Hybrid System, Clontech), which carries a GAL4 activation domain and *LEU2* selection marker. After the LZ was successfully incorporated, the genes for the ArntbHLH or Arnt basic region were inserted into the *EcoR* I and *BamH* I sites of the recombinant pGAD424 (for construction of ArntbHLH, only the gene expressing ArntbHLH was inserted into the original pGAD424 vector).

The recombinant plasmids of these three constructs were transformed into *E. coli* strain SURE (Stop Unwanted Rearrangement Events, Stratagene, La Jolla, CA) by electroporation (Bio-Rad GenePulser XCell electroporation unit), and the cloned insert was sequenced on an ABI (Applied Biosystems) 3730XL 96 capillary sequencer at the DNA Sequencing Facility in the Centre for Applied Genomics, Hospital for Sick Children (Toronto, ON).

### Yeast one-Hybrid assay using the *HIS3* reporter

The Matchmaker One-Hybrid System from Clontech was employed for detection of protein-DNA recognition *in vivo*. Electrocompetent cells of the reporter strain were prepared following a protocol based on the methods described by Suga and Hatakeyama [58], [59]. Yeast cells were grown overnight in YPDA liquid medium (20 g/L Difco peptone, 10 g/L yeast extract, 0.009% adenine hemisulphate). The overnight culture was used to inoculate a new culture that was grown to an OD_600_ over 0.5 (30°C, shaking at 250 rpm). Cells were then harvested by centrifugation (1600g, 5 min, Beckman J2HC high-speed centrifuge) and washed twice with ice-cold H_2_O, followed by one wash with ice-cold 1 M sorbitol and centrifugation again as before. We modified the protocol by the following additional step: the yeast cells were incubated in reducing buffer (1 mM Tris, pH 7.5, 1 mM EDTA, 1 mM LiOAc, 10 mM DTT) at room temperature for 1 hour, followed by three washes of ice-cold sorbitol to improve transformation efficiency. After all washing steps, cells were resuspended in cell resuspension buffer (10 mM 2-[4-(2-hydroxyethyl)-1-piperazinyl] ethanesulfonic acid [HEPES], pH 7.5, 10 mM CaCl_2_, 600 mM sorbitol) to give approximately 5×10^8^ cells/mL, aliquoted (approximately 500 µL cells per tube), and stored at −80°C.

For transformation, 300 ng of each plasmid expressing ArntbHLH-C/EBP, ArntbHLH, or Arnt-C/EBP were electroporated with 40 µL competent reporter-strain cells using a preset program for *S. cerevisiae* (Voltage: 1500 V, Capacitance: 25 µF, Resistance: 200 Ω, 2 mm gap cuvette). The electroporated cells were immediately diluted in 1 mL ice-cold 1 M sorbitol and incubated at room temperature for 30 min. Following incubation, cells were plated on a minimal selective medium lacking leucine and histidine with 10 mM and 20 mM 3-AT. Native MaxbHLHZ and plasmid pGAD424 were transformed as positive and negative controls.

Transformation efficiency (number of colonies/µg plasmid DNA) was calculated using the following formula: [number of colonies×resuspension volume (µL)×dilution factor]/[volume plated (µL)×amount of linearized pGAD424 transformed (µg)]. For supercoiled plamids, the transformation efficiency is generally around 10^5^ colonies per µg plasmid DNA transformed.

### Further testing by *LacZ* reporter

Another reporter strain YM4271[pLacZi/Ebox] was constructed such that four tandem copies of the E-box reside upstream of the *LacZ* gene. This recombinant reporter plasmid was linearized at the *Nco* I site and integrated into the *ura3-52* locus in the genome of *S. cerevisiae* YM4271. The reporter strain was maintained using minimal medium plates lacking uracil. The plasmids for expression of ArntbHLH-C/EBP, ArntbHLH, and Arnt-C/EBP were transformed into integrated reporter strain YM4271[pHISi-1/E-box] by electroporation. Protein:DNA interactions were detected by two commonly used assays based on the *LacZ* reporter: X-gal colony-lift filter assay and *ortho*-nitrophenyl-galactoside (ONPG) liquid assay. These protocols were provided in the Matchmaker One-Hybrid System (Clontech).

In the X-gal colony-lift filter assay, the lysed yeast cells were incubated with X-gal for three hours, and blue color developing after three-hour incubation was not considered to be indicative of positive protein:DNA interactions. For ONPG assays, nine to twelve individual measurements (from three to four separate cell-growth cultures) were used to calculate the β-galactosidase activities for each fusion hybrid. ONPG values are given in dimensionless β-galactosidase units, defined as the amount that hydrolyzes 1 µmol ONPG to *o*-nitrophenol and D-galactose/min•cell [60].

### Fluorescence anisotropy measurements

The genes for ArntbHLH and ArntbHLHZ were reconstructed in codons preferred for bacterial expression and cloned into restriction sites *Nco* I and *Xho* I in pET-28A(+) (Novagen, Mississauga, ON); the genes subcloned from yeast did not express protein even from the *E. coli* Rosetta(DE3)pLysS strain (Novagen), useful for expressing proteins containing codons not optimal for bacterial expression. Even after reconstructing the genes in bacteria-preferred codons, expression was best from the Rosetta strain in comparison to other BL21 derivatives. Proteins were purified by TALON metal ion affinity chromatography (Clontech) and reversed-phase HPLC (Beckman, Fullerton, CA; preparative HPLC traces are shown in Fig. S5, Supporting Information) and identities confirmed by ESI-MS (see ref. 45 for detailed protocols). Protein concentrations were assessed by Tyr absorbance (1 Tyr in Helix 1, absorbance maximum 275–280 nM, ε_275_ = 1405 M^−1^•cm^−1^ per tyrosine) on a Beckman DU 640 UV/vis spectrophotometer.

Compared with the 56-mer Arnt bHLH domain used by Brennan and coworkers, our ArntbHLH derivative contains an additional 18 aa: DQMSNDKERF at the N-terminus, and LEHHHHHH at the C-terminus (Fig. 2) [22]. The N-terminal 10 aa are part of the Arnt N-terminal region, and the C-terminal 8 aa come from the expression vector and contain the 6×His tag.

Fluorescein-labeled E-box and nonspecific oligonucleotides were synthesized by Operon Biotechnologies. The 6-carboxyfluorescein label (6-FAM) was incorporated at the 5′ end of the labeled oligonucleotides, and all oligonucleotides were purified by HPLC. Oligonucleotides were hybridized by heating 10 pmol FAM-oligonucleotide and 15 pmol unlabeled complementary oligonucleotide in annealing buffer (10 mM Tris, pH 7.6, 50 mM NaCl, 1 mM EDTA) at 80°C for 10 min, followed by slow cooling to room temperature over 2 hrs.

Fluorescence was measured on a JY Horiba Fluorolog-3 spectrofluorimeter (University of Toronto) arranged in the L-format (488 nm excitation; 520 nm emission; integration time, 1 sec; band pass, 4 nm). Polarization (P) was measured at each titration point; each value is the average of 10 measurements. 0.9–1.5 µL stock protein solution (1–50 µM monomeric protein) was added per titration point and mixed by pipetting in a total volume of 0.3 mL. The cell (Starna, Atascadero, CA) contained 1 nM DNA duplex in the buffers listed below. Titrations were performed at 22.0±0.2°C. The volume change was kept to <5% of total volume.

Buffers used in anisotropy titrations include the following. *Buffer A*: 4.3 mM Na_2_HPO_4_, pH 7.4, 1.4 mM KH_2_PO_4_, 150 mM NaCl, 2.7 mM KCl, 1 mM EDTA, 800 mM urea, 20% glycerol, 0.1 mg/mL acetylated BSA, and 100 µM bp calf thymus DNA. *Buffer B*: 100 mM Tris, pH 7.4, 150 mM NaCl, 1 mM EDTA, 200 mM guanidine-HCl, 20% glycerol, 0.1 mg/mL acetylated BSA, and 100 µM bp calf thymus DNA. *Buffer C*: 100 mM Tris, pH 7.4, 150 mM NaCl, 1 mM EDTA, and 1.0 µg/mL poly dI-dC. Buffer C is the identical to that used by Brennan and coworkers in their fluorescence anisotropy measurements on the ArntbHLH domain [22]. Buffer B is our modified version of Brennan's buffer containing extra reagents to aid protein stability.

For each data point, the sample was incubated at 4°C overnight followed by at least 20 min at room temperature before measurement; such extensive incubation of the sample after each addition of protein was necessary to minimize protein misfolding and aggregation. We previously have used the temperature-leap tactic to promote and maintain properly folded protein structure [44], [45]. This must be performed after any change in protein concentration and typically involves a 2 hr incubation at 4°C that allows for the slow, proper folding pathway to be populated rather than more rapid, misfolding pathway [44]. However, for the Arnt derivatives, less than overnight incubation at 4°C was insufficient for proper, stable folding.

### Determination of *K*_d_ values

The polarization values were used to calculate apparent dissociation constants using Kaleidagraph 3.6 (Synergy software). Eqn. (1) and the treatment of the calculation of dissociation constants is the same as that used by Brennan and coworkers [22].
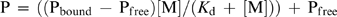
(1)where *K*_d_ corresponds to the apparent monomeric dissociation constant, M is the concentration of monomeric protein, P_free_ is the polarization for free DNA, and P_bound_ is the maximum polarization of specifically bound DNA. Eqn. (1) is used, for the calculated dissociation constants are all at least 40-fold greater than the concentration of labeled DNA duplex; therefore, the concentration of protein bound to DNA is negligible compared with total protein concentration. Only data sets fit to Eqn. (1) with *R* values >0.970 are reported; two independent titrations were performed for each *K*_d_ value presented. *K*_d_ values are given ±SEM (standard error of the mean).

### Circular Dichroism Spectroscopy

Proteins were purified and concentrations were determined by Tyr absorbance, as above. 1 mL samples were prepared with 2 µM ArntbHLH or ArntbHLH-CEBP. Buffers used are as follows: *Buffer D*: 15.08 mM Na_2_HPO_4_, 4.92 mM KH_2_PO_4_, 50 mM NaCl; *Buffer E*: 15.08 mM Na_2_HPO_4_, 4.92 mM KH_2_PO_4_, 50 mM NaCl, 800 mM urea (i.e., Buffer E = Buffer D+800 mM urea); Buffer F: 15.08 mM Na_2_HPO_4_, 4.92 mM KH_2_PO_4_, 50 mM NaCl, 100 µM in bp calf thymus DNA (i.e., Buffer F = Buffer D+100 µM CT DNA). All buffers were pH 7.4. The temperature-leap tactic described above was used to generate functional proteins for CD measurements. Samples, including buffer controls without protein, were prepared and incubated overnight at 4°C, followed by at least 20 min incubation at room temperature. CD was performed on an Aviv 215 spectrometer with a suprasil, 10 mm path-length cell (Hellma, Plainview, NY) at 22°C. Spectra were acquired between 180 and 300 nm at 0.2 nm increments and a sampling time of 0.2 s. Each spectrum was the average of two scans with the average buffer control spectrum subtracted. Data obtained in Buffer D were not smoothed (Fig. 6). Data obtain in Buffers E and F (Fig. S3, Supporting Information) were smoothed using the Aviv 215 software. Protein helix content was calculated by the method of Chau and coworkers [61].

## Supporting Information

Figure S1 Western blot of Y2H. Lanes 1 and 2 are from the same membrane, and lanes 3 and 4 are from the same membrane. Lanes 1 and 3: Precision Plus Protein WesternC standard (Bio- Rad). Lane 2: pGADT7/ArntbHLH ( = Gal4AD+ArntbHLH) supernatant fraction. Lane 4: pGADT7/ArntbHLH ( = Gal4AD+ArntbHLH) pellet fraction. Arrows indicate the bands associated with Gal4AD+ArntbHLH. For experimental details see Materials S1.
(2.73 MB TIF)

Figure S2 URL information. The above URLs provide the pdfs of pGAD424 (Y1H) and pGADT7 (Y2H) vectors from their commercial suppliers.
(0.72 MB TIF)

Figure S3 Additional fluorescence anisotropy titration binding isotherms. The second pair of isotherms of ArntbHLH-C/EBP (A, •, solid blue line) and ArntbHLH (B, Δ, dashed red line) binding to the E-box in Buffer B (100 mM Tris, pH 7.4, 150 mM NaCl, 1 mM EDTA, 200 mM guanidine-HCl, 20% glycerol, 0.1 mg/mL acetylated BSA, and 100 µM bp calf thymus DNA). The first pair of isotherms is shown in Figure 5 of the manuscript. (C) The pair of binding isotherms of ArntbHLH (Δ, dashed red line) and ArntbHLH-C/EBP (•, solid blue line) binding to the E-box in Buffer C (100 mM Tris, pH 7.4, 150 mM NaCl, 1 mM EDTA, and 1.0 µg/mL poly dI-dC). Buffer C is identical to that used by Brennan and coworkers in their fluorescence anisotropy measurements on the ArntbHLH domain [Huffman JL, Mokashi A, Bachinger HP, Brennan RG (2001) The Basic Helix-Loop-Helix Domain of the Aryl Hydrocarbon Receptor Nuclear Transporter (ARNT) Can Oligomerize and Bind E-box DNA Specifically. J Biol Chem 276: 40537–40544.]. For the Kd values obtained with these data, only one isotherm was used (in contrast to the Kd values obtained in Buffer B from two separate isotherms), and therefore, these values are not given with SEM and are presented as approximate. The plateaus of these isotherms were also not achieved, and therefore, the Kd values generated are not as accurate as those in Buffer B.
(2.56 MB TIF)

Figure S4 Circular dichroism. Spectra of ArntbHLH (red line) and ArntbHLH-C/EBP (blue line). Samples contained 2 µM ArntbHLH or ArntbHLH-CEBP. Buffers used are as follows: *Buffer D:* 15.08 mM Na_2_HPO_4_, 4.92 mM KH_2_PO_4_, 50 mM NaCl; *Buffer E:* 15.08 mM Na_2_HPO_4_, 4.92 mM KH_2_PO_4_, 50 mM NaCl, 800 mM urea (i.e., Buffer E = Buffer D+800 mM urea); Buffer F: 15.08 mM Na_2_HPO_4_, 4.92 mM KH_2_PO_4_, 50 mM NaCl, 100 µM in bp calf thymus DNA (i.e., Buffer F = Buffer D+100 µM CT DNA). All buffers were pH 7.4. Left. Data obtained in Buffer E. ArntbHLH-C/EBP shows 49% helicity, as measured at 222 nm. Right. Data obtained in Buffer F. ArntbHLH-C/EBP shows 36% helicity and ArntbHLH shows 29% helicity, as measured at 222 nm. Each spectrum was averaged twice, and curves were subjected to smoothing (in contrast, the curves in Fig. 6 of the manuscript were *not smoothed*, but with urea or CT DNA, much more noise arose). The buffer control was subtracted from each protein spectrum. Percent helix content was determined assuming only helical content at 222 nm using the equation H = θ_222_/[θ_H222_∞(1-k_222_/n)] where H is percent helicity, θ_222_ is the mean residue elipticity at 222 nM, θ_H222_∞ is the reference value for a helix of infinite length, k_222_ is a wavelength dependant constant and n is the number of amino acids in the protein [Chen Y-H, Yang JT, Chau KH (1974) Determination of the Helix and β Form of Proteins in Aqueous Solution by Circular Dichroism. Biochemistry 13: 3350–3359.].
(1.78 MB TIF)

Figure S5 Preparative HPLC traces of protein used in fluorescence anisotropy analysis. Traces above show protein after the first purification by immobilized metal-ion affinity chromatography with TALON (Clontech), which significantly purifies the proteins. The second purification is with HPLC. In the above preparative traces, only the major peak is collected, so the shoulders are removed. ESI-MS confirms the identity of the major peak as being either (A) ArntbHLH or (B) ArntbHLH-C/EBP, both monitored at 220 nm. Thus, a high level of purity of proteins is used in the FA assays. Each protein was purified by HPLC (Beckman System Gold) on a semipreparative reversed-phase C4 column (Vydac, Hesperia, CA) with a gradient of acetonitrile-water plus 0.05% trifluoroacetic acid (v/v) at flow rate 4 mL/min; the gradient started at 10–25% acetonitrile over 15 min, followed by 25–55% acetonitrile over 60 min.
(1.13 MB TIF)

Materials S1 Experimental Details for Y2H
(0.09 MB PDF)

## References

[1] Struhl K (1989). Helix-turn-helix, zinc-finger, and leucine-zipper motifs for eucaryotic transcriptional regulatory proteins.. Trends Biochem Sci.

[2] Landschulz WH, Johnson PF, McKnight SL (1988). The Leucine Zipper: A Hypothetical Structure Common to a New Class of DNA Binding Proteins.. Science.

[3] König P, Richmond TJ (1993). The X-ray structure of the GCN4-bZIP bound to ATF/CREB site DNA shows the complex depends on DNA flexibility.. J Mol Biol.

[4] Ellenberger TE, Brandl CJ, Struhl K, Harrison SC (1992). The GCN4 basic region leucine zipper binds DNA as a dimer of uninterrupted α helices: Crystal stucture of the protein-DNA complex.. Cell.

[5] Keller W, König P, Richmond TJ (1995). Crystal structure of a bZIP/DNA Complex at 2.2 Å: Determinants of DNA specific recognition.. J Mol Biol.

[6] Glover JNM, Harrison SC (1995). Crystal structure of the heterodimeric bZIP transcription factor c-Fos-c-Jun bound to DNA.. Nature.

[7] Ferre-D'Amare AR, Prendergast GC, Ziff EB, Burley SK (1993). Recognition by Max of its cognate DNA through a dimeric b/HLH/Z domain.. Nature.

[8] Brownlie P, Ceska TA, Lamers M, Romier C, Stier G (1997). The crystal structure of an intact human Max-DNA complex: new insights into mechanisms of transcriptional control.. Structure.

[9] Nair SK, Burley SK (2003). X-Ray Structure of Myc-Max and Mad-Max Recognizing DNA: Molecular Bases of Regulation by Proto-Oncogenic Transcription Factors.. Cell.

[10] Kewley RJ, Whitelaw ML, Chapman-Smith A (2004). The mammalian basic helix-loop-helix/PAS family of transcriptional regulators.. Int J Biochem Cell Biol.

[11] Massari ME, Murre C (2000). Helix-Loop-Helix Proteins: Regulators of Transcription in Eucaryotic Organisms.. Mol Cell Biol.

[12] Jones S (2004). An overview of the basic helix-loop-helix proteins.. Genome Biol.

[13] Gardner L, Lee L, Dang C, Bertino JR (2002). The c-Myc Oncogenic Transcription Factor.. Encyclopedia of Cancer.

[14] Hoffman EC, Reyes H, Chu F, Sander F, Conley LH (1991). Cloning of a Factor Required for Activity of the Ah (Dioxin) Receptor.. Science.

[15] Reyes H, Reisz-Porszasz S, Hankinson O (1992). Identification of the Ah Receptor Nuclear Translocator Protein (Arnt) as a Component of the DNA Binding Form of the Ah Receptor.. Science.

[16] Schmid T, Zhou J, Brüne B (2004). HIF-1 and p53: communication of transcription factors under hypoxia.. J Cell Mol Med.

[17] Swanson HI, Yang J-H (1999). Specificity of DNA binding of the c-Myc/Max and ARNT/ARNT dimers at the CACGTG recognition site.. Nucl Acid Res.

[18] Arpiainen S, Lämsä V, Pelkonen O, Yim SH, Gonzalez FJ (2007). Aryl Hydrocarbon Receptor Nuclear Translocator and Upstream Stimulatory Factor Regulate Cytochrome P450 2a5 Transcription through a Common E-box Site.. J Mol Biol.

[19] Blackwood EM, Eisenman RN (1991). Max: A Helix-Loop-Helix Zipper Protein That Forms a Sequence-Specific DNA-Binding Complex with Myc.. Science.

[20] Swanson HI (2002). DNA binding and protein interactions of the AHR/ARNT heterodimer that facilitate gene activation.. Chem Biol Interact.

[21] Chapman-Smith A, Whitelaw ML (2006). Novel DNA Binding by a Basic Helix-Loop-Helix Protein: The Role of the Dioxin Receptor PAS Domain.. J Biol Chem.

[22] Huffman JL, Mokashi A, Bachinger HP, Brennan RG (2001). The Basic Helix-Loop-Helix Domain of the Aryl Hydrocarbon Receptor Nuclear Transporter (ARNT) Can Oligomerize and Bind E-box DNA Specifically.. J Biol Chem.

[23] Agre P, Johnson PF, McKnight SL (1989). Cognate DNA binding specificity retained after leucine zipper exchange between GCN4 and C/EBP.. Science.

[24] Lajmi AR, Lovrencic ME, Wallace TR, Thomlinson RR, Shin JA (2000). Minimalist, Alanine-based, Helical Protein Dimers Bind to Specific DNA Sites.. J Am Chem Soc.

[25] Sellers JW, Struhl K (1989). Changing Fos oncoprotein to a Jun-independent DNA-binding protein with GCN4 dimerization specificity by swapping ‘leucine zippers’.. Nature.

[26] Yin X, Grove L, Prochownik EV (1998). Lack of transcriptional repression by max homodimers.. Oncogene.

[27] Chapman-Smith A, Lutwyche JK, Whitelaw ML (2004). Contribution of the Per/Arnt/Sim (PAS) Domains to DNA Binding by the Basic Helix-Loop-Helix PAS Transcriptional Regulators.. J Biol Chem.

[28] O'Neil KT, DeGrado WF (1990). A thermodynamic scale for the helix-forming tendencies of the commonly occurring amino acids.. Science.

[29] Ma PCM, Rould MA, Weintraub H, Pabo CO (1994). Crystal Structure of MyoD bHLH Domain-DNA Complex: Perspectives on DNA Recognition and Implications for Transcriptional Activation.. Cell.

[30] Wang MM, Reed RR (1993). Molecular cloning of the olfactory neuronal transcription factor Olf-1 by genetic selection in yeast.. Nature.

[31] Estojak J, Brent R, Golemis EA (1995). Correlation of two-hybrid affinity data with in vitro measurements.. Mol Cell Biol.

[32] Vidal M, Legrain P (1999). Yeast forward and reverse ‘n’-hybrid systems.. Nucl Acid Res.

[33] Serebriiskii IG, Golemis EA (2000). Uses of lacZ to Study Gene Function: Evaluation of b-Galactosidase Assays Employed in the Yeast Two-Hybrid System.. Anal Biochem.

[34] Möckli N, Auerbach D (2004). Quantitative b-galactosidase assay suitable for high-throughput applications in the yeast two-hybrid system.. BioTechniques.

[35] Reisz-Porszasz S, Probst MR, Fukunaga BN, Hankinson O (1994). Identification of Functional Domains of the Aryl Hydrocarbon Receptor Nuclear Translocator Protein (ARNT).. Mol Cell Biol.

[36] Clontech (2001). Yeast Protocols Handbook..

[37] Lee D, Seol W, Kim J-S (2003). Custom DNA-binding proteins and artificial transcription factors.. Curr Topics Med Chem.

[38] Blancafort P, Segal DJ, Barbas CF (2004). Designing transcription factor architectures for drug discovery.. Mol Pharm.

[39] Dervan PB, Doss RM, Marques MA (2005). Programmable DNA binding oligomers for control of transcription.. Curr Med Chem-Anti-Cancer Agents.

[40] Nagaoka M, Sugiura Y (2000). Artificial zinc finger peptides: creation, DNA recognition, and gene regulation.. J Inorg Biochem.

[41] Dreier B, Fuller RP, Segal DJ, Lund CV, Blancafort P (2005). Development of zinc finger domains for recognition of the 5′-CNN-3′ family DNA sequences and their use in the construction of artificial transcription factors.. J Biol Chem.

[42] Dhanasekaran M, Negi S, Sugiura Y (2006). Designer zinc finger proteins: tools for creating artificial DNA-binding functional proteins.. Acc Chem Res.

[43] Mandell JG, Barbas CF (2006). Zinc Finger Tools: custom DNA-binding domains for transcription factors and nucleases.. Nucl Acid Res 34 (Web Server issue).

[44] Xie Y, Wetlaufer DB (1996). Control of aggregation in protein refolding: The temperature-leap tactic.. Prot Sci.

[45] Lajmi AR, Wallace TR, Shin JA (2000). Short, Hydrophobic, Alanine-based Proteins Based on the bZIP Motif: Overcoming Inclusion Body Formation and Protein Aggregation During Overexpression, Purification, and Renaturation.. Prot Exp Purif.

[46] Jung KC, Rhee HS, Park CH, Yang C-H (2005). Determination of the dissociation constants for recombinant c-Myc, Max, and DNA complexes: The inhibitory effect of linoleic acid on the DNA-binding step.. Biochem Biophys Res Comm.

[47] Hu J, Banerjee A, Goss DJ (2005). Assembly of b/HLH/Z Proteins c-Myc, Max, and Mad1 with Cognate DNA: Importance of Protein-Protein and Protein-DNA Interactions.. Biochemistry.

[48] Meier-Andrejszki L, Bjelic S, Naud J-F, Lavigne P, Jelesarov I (2007). Thermodynamics of b-HLH-LZ Protein Binding to DNA: The Energetic Importance of Protein-DNA Contacts in Site-Specific E-Box Recognition by the Complete Gene Product of the Max p21 Transcription Factor.. Biochemistry.

[49] Walhout AJM, Sordella R, Lu XW, Hartley JL, Temple GF (2000). Protein interaction mapping in C-elegans using proteins involved in vulval development.. Science.

[50] Saudek V, Pasley HS, Gibson T, Gausepohl H, Frank R (1991). Solution structure of the basic region from the transcriptional activator GCN4.. Biochemistry.

[51] O'Neil KT, Shuman JD, Ampe C, DeGrado WF (1991). DNA-induced increase in the α-helical content of C/EBP and GCN4.. Biochemistry.

[52] Weiss MA, Ellenberger TE, Wobbe CR, Lee JP, Harrison SC (1990). Folding transition in the DNA-binding domain of GCN4 on specific binding to DNA.. Nature.

[53] Shin JA (1997). Specific DNA binding peptide derivatized solid support.. Bioorg Med Chem Lett.

[54] Hollenbeck JJ, Oakley MG (2000). GCN4 Binds with High Affinity to DNA Sequences Containing a Single Consensus Half-Site.. Biochemistry.

[55] Bracken C, Carr PA, Cavanagh J, Palmer AG (1999). Temperature Dependence of Intramolecular Dynamics of the Basic Leucine Zipper of GCN4: Implications for the Entropy of Association with DNA.. J Mol Biol.

[56] Fersht AR (2008). From the first protein structures to our current knowledge of protein folding: delights and scepticisms.. Nature Rev.

[57] Wu G, Wolf JB, Ibrahim AF, Vadasz S, Gunasinghe M (2006). Simplified gene synthesis: A one-step approach to PCR-based gene construction.. J Biotechnol.

[58] Suga M, Hatakeyama T (2001). High efficiency transformation of Schizosaccharomyces pombe pretreated with thiol compounds by electroporation.. Yeast.

[59] Suga M, Hatakeyama T (2003). High efficiency electroporation by freezing intact cells with addition of calcium.. Curr Genet.

[60] Miller JH (1972). Experiments in Molecular Genetics.

[61] Chen Y-H, Yang JT, Chau KH (1974). Determination of the Helix and β Form of Proteins in Aqueous Solution by Circular Dichroism.. Biochemistry.

[62] Yildiz Ö, Doi M, Yujnovsky I, Cardone L, Berndt A (2005). Crystal Structure and Interactions of the PAS Repeat Region of the Drosophila Clock Protein PERIOD.. Mol Cell.

