# Supplementary figures and images for: Hybrids of the bHLH and bZIP Protein Motifs Display Different DNA-Binding Activities In Vivo vs. In Vitro
Source: PLoS One. 2008 Oct 24;3(10):e3514. doi: 10.1371/journal.pone.0003514 (PMC2568859; doi:10.1371/journal.pone.0003514)

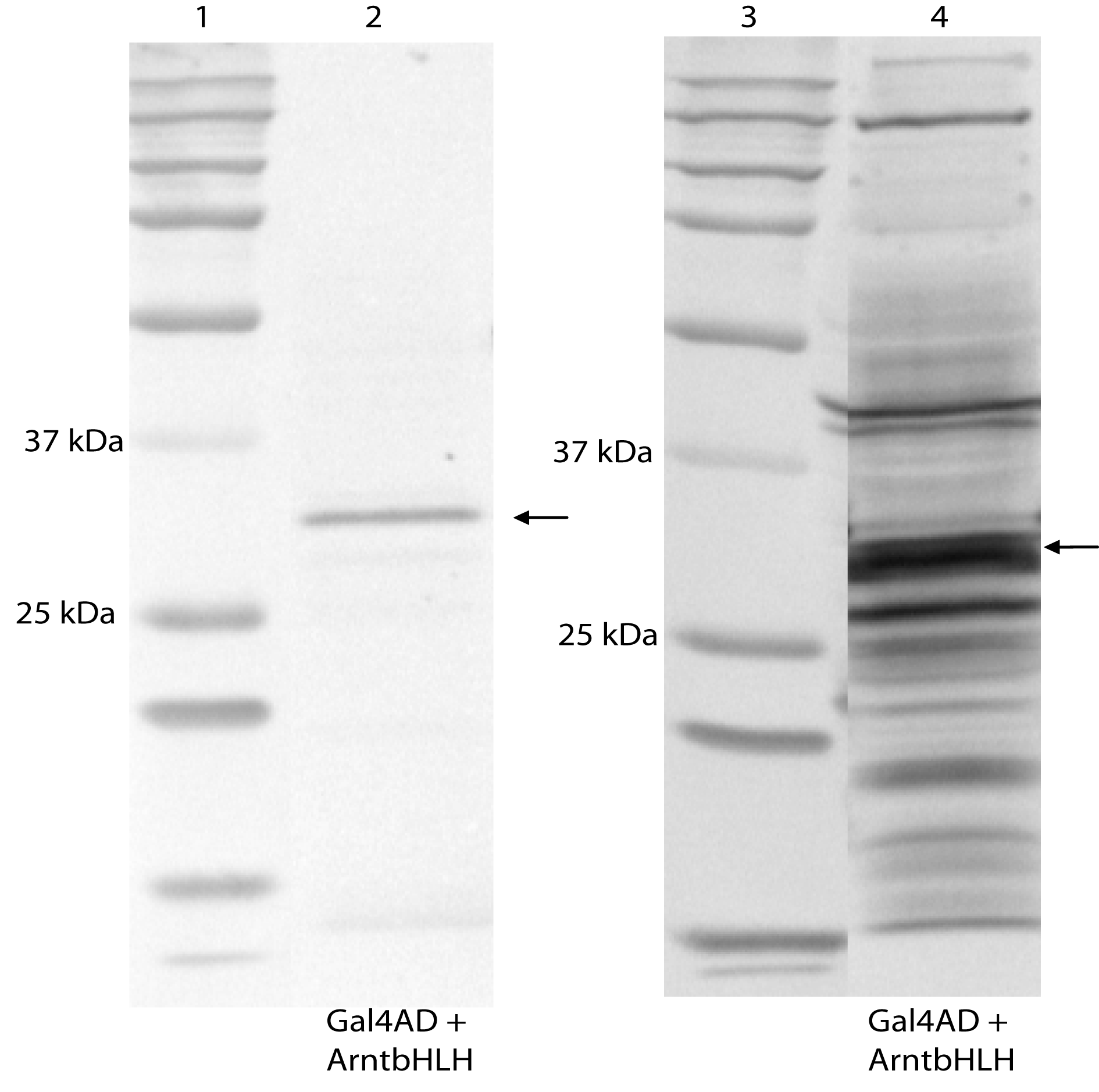

Supplement: Figure S1 — Western blot of Y2H. Lanes 1 and 2 are from the same membrane, and lanes 3 and 4 are from the same membrane. Lanes 1 and 3: Precision Plus Protein WesternC standard (Bio- Rad). Lane 2: pGADT7/ArntbHLH ( = Gal4AD+ArntbHLH) supernatant fraction. Lane 4: pGADT7/ArntbHLH ( = Gal4AD+ArntbHLH) pellet fraction. Arrows indicate the bands associated with Gal4AD+ArntbHLH. For experimental details see Materials S1. (2.73 MB TIF) [file pone.0003514.s001.tif]

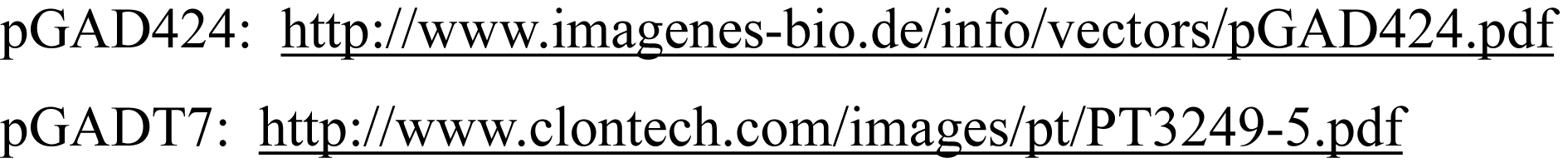

Supplement: Figure S2 — URL information. The above URLs provide the pdfs of pGAD424 (Y1H) and pGADT7 (Y2H) vectors from their commercial suppliers. (0.72 MB TIF) [file pone.0003514.s002.tif]

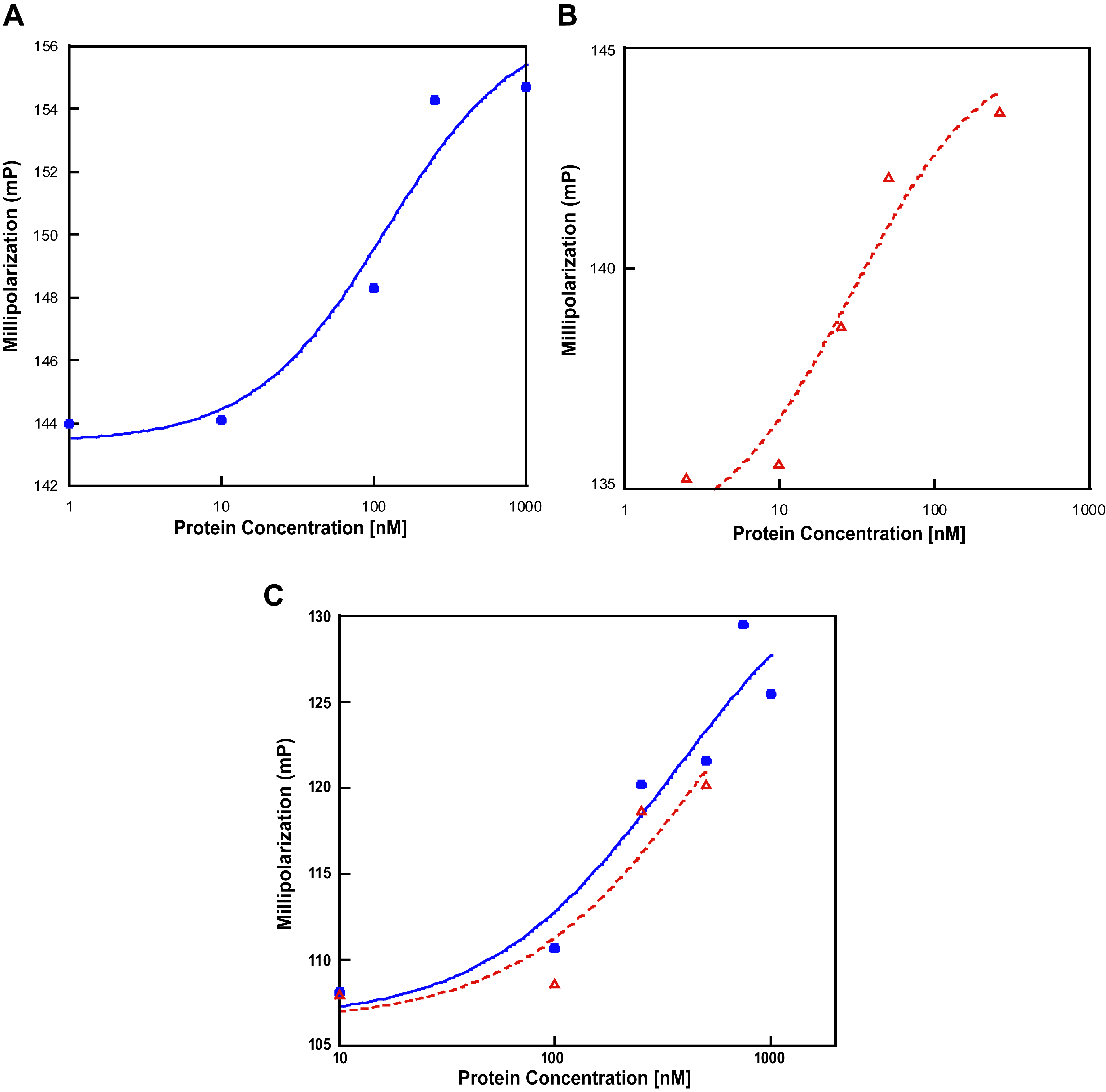

Supplement: Figure S3 — Additional fluorescence anisotropy titration binding isotherms. The second pair of isotherms of ArntbHLH-C/EBP (A, •, solid blue line) and ArntbHLH (B, Δ, dashed red line) binding to the E-box in Buffer B (100 mM Tris, pH 7.4, 150 mM NaCl, 1 mM EDTA, 200 mM guanidine-HCl, 20% glycerol, 0.1 mg/mL acetylated BSA, and 100 µM bp calf thymus DNA). The first pair of isotherms is shown in Figure 5 of the manuscript. (C) The pair of binding isotherms of ArntbHLH (Δ, dashed red line) and ArntbHLH-C/EBP (•, solid blue line) binding to the E-box in Buffer C (100 mM Tris, pH 7.4, 150 mM NaCl, 1 mM EDTA, and 1.0 µg/mL poly dI-dC). Buffer C is identical to that used by Brennan and coworkers in their fluorescence anisotropy measurements on the ArntbHLH domain [Huffman JL, Mokashi A, Bachinger HP, Brennan RG (2001) The Basic Helix-Loop-Helix Domain of the Aryl Hydrocarbon Receptor Nuclear Transporter (ARNT) Can Oligomerize and Bind E-box DNA Specifically. J Biol Chem 276: 40537–40544.]. For the Kd values obtained with these data, only one isotherm was used (in contrast to the Kd values obtained in Buffer B from two separate isotherms), and therefore, these values are not given with SEM and are presented as approximate. The plateaus of these isotherms were also not achieved, and therefore, the Kd values generated are not as accurate as those in Buffer B. (2.56 MB TIF) [file pone.0003514.s003.tif]

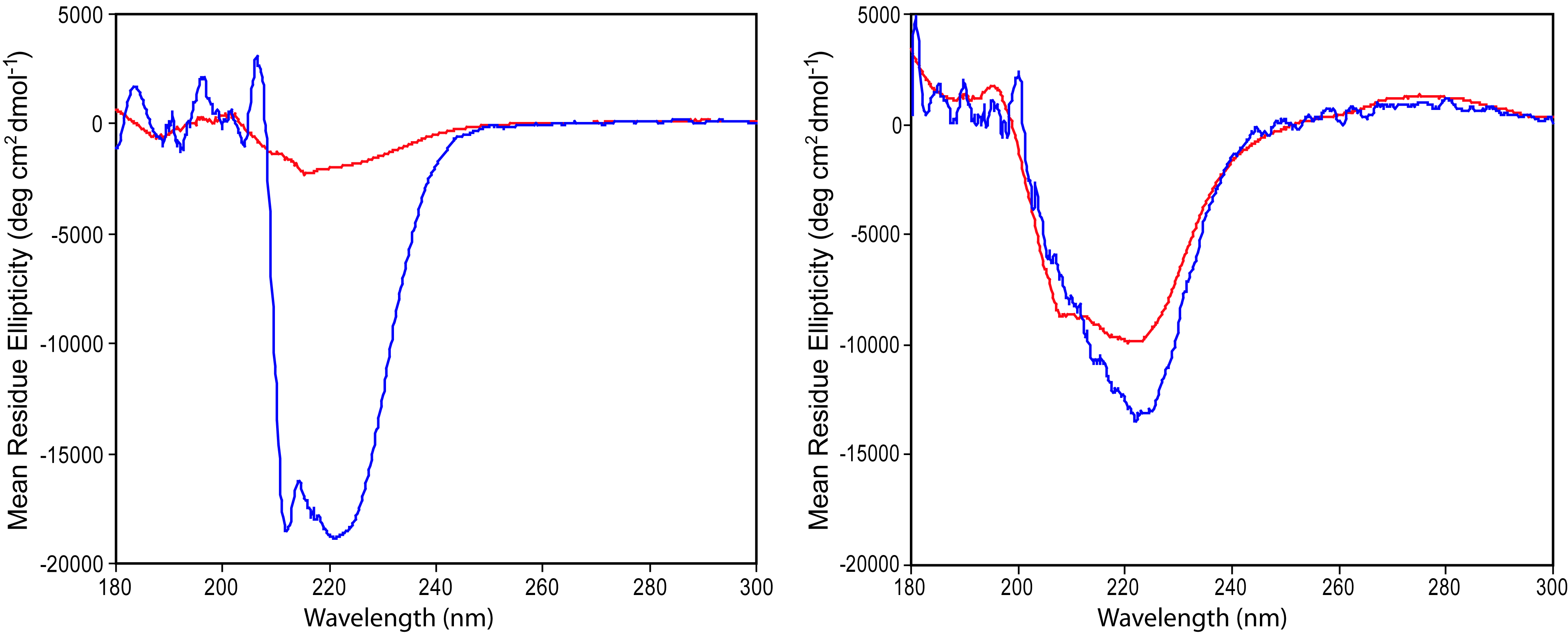

Supplement: Figure S4 — Circular dichroism. Spectra of ArntbHLH (red line) and ArntbHLH-C/EBP (blue line). Samples contained 2 µM ArntbHLH or ArntbHLH-CEBP. Buffers used are as follows: Buffer D: 15.08 mM Na2HPO4, 4.92 mM KH2PO4, 50 mM NaCl; Buffer E: 15.08 mM Na2HPO4, 4.92 mM KH2PO4, 50 mM NaCl, 800 mM urea (i.e., Buffer E = Buffer D+800 mM urea); Buffer F: 15.08 mM Na2HPO4, 4.92 mM KH2PO4, 50 mM NaCl, 100 µM in bp calf thymus DNA (i.e., Buffer F = Buffer D+100 µM CT DNA). All buffers were pH 7.4. Left. Data obtained in Buffer E. ArntbHLH-C/EBP shows 49% helicity, as measured at 222 nm. Right. Data obtained in Buffer F. ArntbHLH-C/EBP shows 36% helicity and ArntbHLH shows 29% helicity, as measured at 222 nm. Each spectrum was averaged twice, and curves were subjected to smoothing (in contrast, the curves in Fig. 6 of the manuscript were not smoothed, but with urea or CT DNA, much more noise arose). The buffer control was subtracted from each protein spectrum. Percent helix content was determined assuming only helical content at 222 nm using the equation H = θ222/[θH222∞(1-k222/n)] where H is percent helicity, θ222 is the mean residue elipticity at 222 nM, θH222∞ is the reference value for a helix of infinite length, k222 is a wavelength dependant constant and n is the number of amino acids in the protein [Chen Y-H, Yang JT, Chau KH (1974) Determination of the Helix and β Form of Proteins in Aqueous Solution by Circular Dichroism. Biochemistry 13: 3350–3359.]. (1.78 MB TIF) [file pone.0003514.s004.tif]

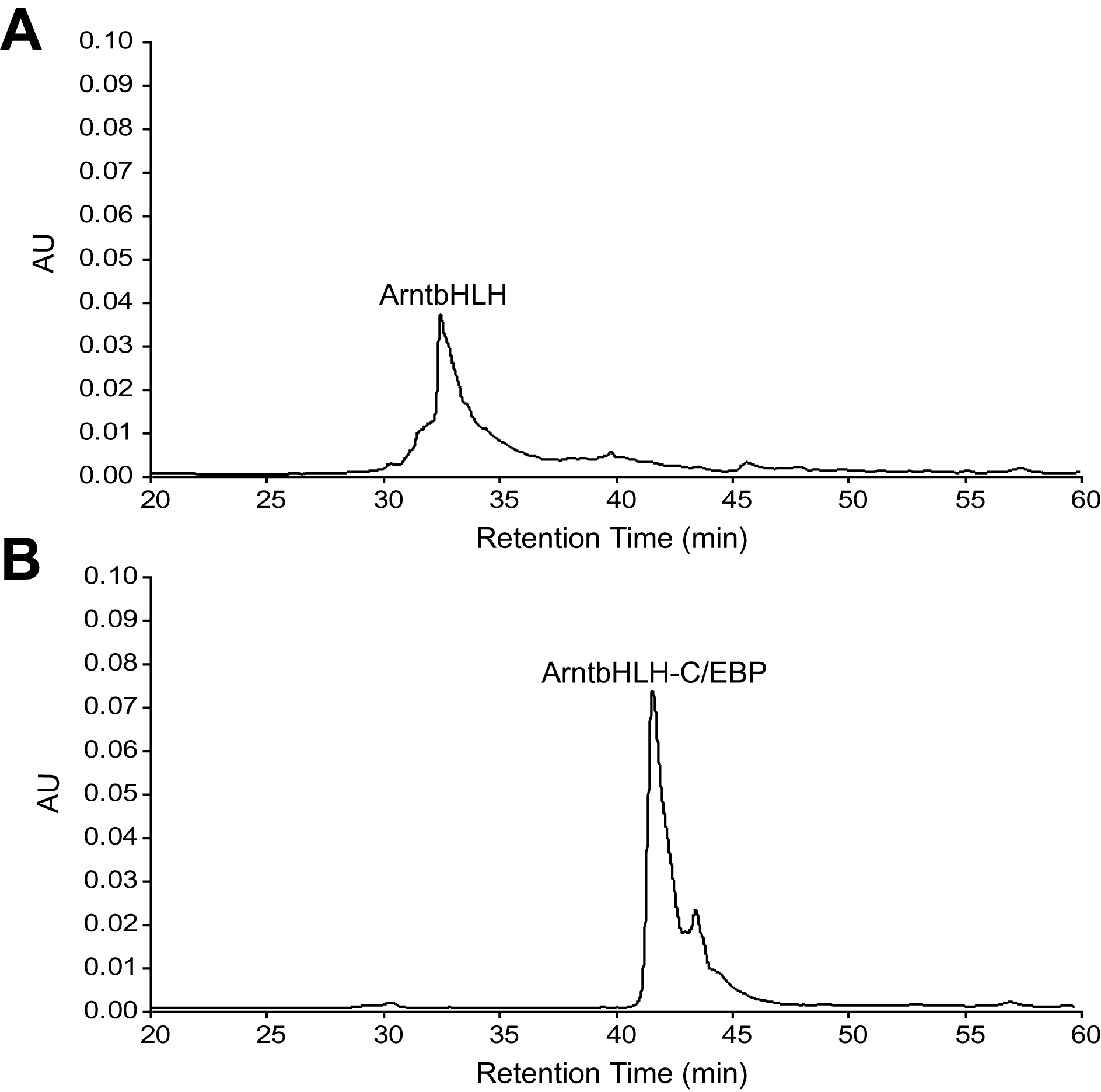

Supplement: Figure S5 — Preparative HPLC traces of protein used in fluorescence anisotropy analysis. Traces above show protein after the first purification by immobilized metal-ion affinity chromatography with TALON (Clontech), which significantly purifies the proteins. The second purification is with HPLC. In the above preparative traces, only the major peak is collected, so the shoulders are removed. ESI-MS confirms the identity of the major peak as being either (A) ArntbHLH or (B) ArntbHLH-C/EBP, both monitored at 220 nm. Thus, a high level of purity of proteins is used in the FA assays. Each protein was purified by HPLC (Beckman System Gold) on a semipreparative reversed-phase C4 column (Vydac, Hesperia, CA) with a gradient of acetonitrile-water plus 0.05% trifluoroacetic acid (v/v) at flow rate 4 mL/min; the gradient started at 10–25% acetonitrile over 15 min, followed by 25–55% acetonitrile over 60 min. (1.13 MB TIF) [file pone.0003514.s005.tif]
