# Supplementary material for: Hybrids of the bHLH and bZIP Protein Motifs Display Different DNA-Binding Activities In Vivo vs. In Vitro
Source: PLoS One. 2008 Oct 24;3(10):e3514. doi: 10.1371/journal.pone.0003514 (PMC2568859; doi:10.1371/journal.pone.0003514)
Supplement: Materials S1 — Experimental Details for Y2H (0.09 MB PDF) [file pone.0003514.s006.pdf]

Experimental Details for Y2H

pGADT7/ArntbHLH

The ArntbHLH fragment was amplified from NArnt cDNA [1] with oligonucleotides 5'-ACT**GAATTC**AGCTCTGCGGATAAAGAGAG-3' and 5'-ATA**CTGCAG**GGATGTGTTGCCAGTTCCCC-3' and inserted between the *EcoR* I and *Pst* I sites of pGBKT7 to generate pGBKT7/ArntbHLH; the *EcoR* I/*Xho* I-digested ArntbHLH fragment described above was inserted into these restriction sites in pGADT7 to generate pGADT7/ArntbHLH. This plasmid encoded the Gal4 activation domain linked to aa 82-149 of human Arnt isoform 1 with a HA tag between the two sequences. This plasmid was transformed into yeast strain AH109 by the TRAFO method [2], along with pGBKT7/AhRbHLH, encoding the Gal4 DNA-binding domain attached to aa 20-90 of human AhR. The transformed cells were plated on SD/–L/–W plates and incubated at 30 °C.

Western Blot

Yeast cells were grown overnight in SD/-H/-L until they reached OD_{600} 1.0-1.3. They were transferred to YPDA with an initial OD_{600} ~0.2 and grown to OD_{600} 0.60-0.64. Cells were then harvested by centrifugation for 5 min at 3300 rpm and resuspended in 100 μL cold lysis buffer (50 mM Tris-HCl, pH 8.0, 150 mM NaCl, 1% Nonidet P-40, 0.5% sodium deoxycholate, 0.1% SDS, and 0.02% sodium azide) containing freshly added protease inhibitors [1 mM phenyl-methylsulfonyl fluoride (PMSF), 25 ng/mL pepstatin]. The suspensions were incubated on ice for 5 min and then subjected to 3 rounds of sonication for 15 sec each. Lysates were again incubated on ice for 5 min and then centrifuged for 10 min at 4 °C. The supernatant was transferred to a new tube and the pellet was resuspended in 100 μL 3M urea and incubated at room temperature for 3 min. 100 μL of SDS-PAGE sample buffer (50 mM Tris-HCl, pH 6.8, 100 mM DTT, 2% SDS, 10% glycerol, and 0.1% bromophenol blue) were added to both supernatant and pellet, and the samples were boiled for 10 min. Samples were stored at -80 °C.

15 μL of samples were loaded on a 15% SDS-PAGE (Tris-Glycine) gel and run at 100 V for 90 min in SDS-PAGE Running Buffer (25 mM Tris base, 192 mM glycine, 0.5% w/v SDS), then transferred by tank electroblotting to a nitrocellulose membrane at 100 V for 75 min in cold transfer buffer (25 mM glycine, 192 mM Tris base, 20% v/v methanol). The membrane was washed in TBS (10 mM Tris-HCl, pH 7.5, 150 mM NaCl) for 10 min, and washed twice in TBS-T (20 mM Tris-HCl, pH 7.5, 500 mM NaCl, 0.05% v/v Tween-20) for 10 min each. The

membrane was blocked overnight in TBS-T containing 5% non-fat skim milk powder at room temperature with shaking. The membrane was washed twice in TBS-T for 10 min each, then once in TBS for 10 min. The primary antibody, monoclonal HA.11 (Covance, Princeton, NJ) was diluted 1000-fold in TBS and the membrane was incubated for 2 hrs at room temperature, followed by washing as before, and incubation with the secondary antibody (goat anti-mouse IgG-HRP conjugate, Santa Cruz Biotechnology, Santa Cruz, CA) for 1 hr at room temperature. The membrane was washed again, as before, and the protein bands were visualized using the ECL Plus Western Blotting Detection System (Amersham Biosciences, Piscataway, NJ) on a Molecular Dynamics Storm 840 PhosphorImager System. Images were prepared using Adobe Photoshop CS and Adobe Illustrator CS.

The gel was washed in water, post-transfer, for 10 min, then stained with Coomassie Brilliant Blue (0.25% w/v in 45.5% v/v ethanol, 9% v/v glacial acetic acid) for 30 min, and de-stained in 10% v/v acetic acid until the desired color was reached.

REFERENCES

1. Chen G, Shin JA (2008) AhR/Arnt:XRE interaction: turning false negatives into true positives in the modified yeast one-hybrid assay. Analytical Biochemistry, in press.
2. Dohmen RJ, Strasser AWM, Höner CB, Hollenberg CP (1991) An efficient transformation procedure enabling long-term storage of competent cells of various yeast genera. Yeast 7: 691-692.
